# Correlation does not equal causation: the imperative of causal inference in machine learning models for immunotherapy

**DOI:** 10.3389/fimmu.2025.1630781

**Published:** 2025-09-17

**Authors:** Jia-Wen Wang, Meng Meng, Mu-Wei Dai, Ping Liang, Juan Hou

**Affiliations:** ^1^ Department of Orthopedics, The Fourth Hospital of Hebei Medical University, Shijiazhuang, Hebei, China; ^2^ Department of Pharmacy, the Fourth Hospital of Hebei Medical University, Shijiazhuang, Hebei, China

**Keywords:** causal inference, machine learning, immunotherapy, immune checkpoint inhibitors, confounding bias, treatment effect estimation, multimodal data integration, precision medicine

## Abstract

Machine learning (ML) has played a crucial role in advancing precision immunotherapy by integrating multi-omics data to identify biomarkers and predict therapeutic responses. However, a prevalent methodological flaw persists in immunological studies—an overreliance on correlation-based analysis while neglecting causal inference. Traditional ML models struggle to capture the intricate dynamics of immune interactions and often function as “black boxes.” A systematic review of 90 studies on immune checkpoint inhibitors revealed that despite employing ML or deep learning techniques, none incorporated causal inference. Similarly, all 36 retrospective studies modeling melanoma exhibited the same limitation. This “knowledge–practice gap” highlights a disconnect: although researchers acknowledge that correlation does not imply causation, causal inference is often omitted in practice. Recent advances in causal ML, like Targeted-BEHRT, CIMLA, and CURE, offer promising solutions. These models can distinguish genuine causal relationships from spurious correlations, integrate multimodal data—including imaging, genomics, and clinical records—and control for unmeasured confounders, thereby enhancing model interpretability and clinical applicability. Nevertheless, practical implementation still faces major challenges, including poor data quality, algorithmic opacity, methodological complexity, and interdisciplinary communication barriers. To bridge these gaps, future efforts must focus on advancing research in causal ML, developing platforms such as the Perturbation Cell Atlas and federated causal learning frameworks, and fostering interdisciplinary training programs. These efforts will be essential to translating causal ML from theoretical innovation to clinical reality in the next 5-10 years—representing not only a methodological upgrade, but also a paradigm shift in immunotherapy research and clinical decision-making.

## 1 Introduction

Machine learning (ML) technologies have played a pivotal role in advancing precision immunotherapy by integrating multi-omics data to identify biomarkers, predict treatment responses, discover novel therapeutic targets (1, 2), characterize the tumor microenvironment, and optimize patient stratification. These predictive models have greatly enhanced clinical decision-making capabilities (3, 4). However, the application of ML in immunology has increasingly come under scrutiny. Traditional models often fail to capture the complexity of immune interactions (5), suffer from the “black-box” nature of deep learning (6), and lack standardized data preprocessing protocols (7).

Despite broad recognition that “correlation ≠ causation” is a fundamental statistical principle, this distinction is frequently overlooked in practice. A systematic review of 90 studies on immune checkpoint inhibitors (ICIs) revealed that while 72% employed traditional ML and 22% used deep learning, none incorporated causal inference. Consequently, these models were not included in phase III clinical trial designs or referenced in major clinical guidelines (8). This phenomenon is not isolated: a parallel analysis of 36 melanoma prediction models showed all studies were retrospective correlation-based analyses, with none applying causal inference. As a result, PROBAST evaluations rated them as having moderate to high bias, limiting their translational utility and clinical applicability (9, 10).

This disconnect between knowledge and practice highlights a broader issue in immunology research—an overreliance on digital correlations. Researchers may acknowledge the importance of causality but are deterred from applying causal frameworks due to the intrinsic complexity of immunological data. High-dimensional, noisy, and temporally dynamic immune responses, combined with treatment-induced nonlinear effects and substantial interindividual heterogeneity (across genotype, phenotype, and microenvironment), pose significant challenges to conventional causal inference methods (11–14).

Fortunately, recent methodological advances have made the integration of causal inference and ML increasingly feasible. For example, the Targeted-BEHRT model combines transformer architecture with doubly robust estimation to infer long-term treatment effects from longitudinal, high-dimensional data (15). Causal network models incorporating selection diagrams, missingness graphs, and structure discovery techniques outperform standard ML in risk evaluation and adverse event prediction for immunotherapies (16). CIMLA exhibits exceptional robustness to confounding in gene regulatory network analysis, offering insights into tumor immune regulation (17). CURE, leveraging large-scale pretraining, improves treatment effect estimation with gains of ~4% in AUC and ~7% in precision-recall performance over traditional methods (18). Causal-stonet handles multimodal and incomplete datasets effectively, crucial for big-data immunology research (19). LingAM-based causal discovery models have demonstrated high accuracy (84.84% with logistic regression; 84.83% with deep learning) and can directly identify causative factors, significantly improving reliability in immunological studies (20).

These innovations represent a confluence of causal reasoning and machine learning methodologies (21), which are now being increasingly applied in immunology research (22, 23). They help reveal true causal relationships, mitigate confounding (both observed and unobserved), enhance model interpretability and robustness (24), and integrate heterogeneous data types including genomics, proteomics, clinical phenotypes, and medical imaging (25, 26). Ultimately, they enable the construction of more realistic models with superior generalizability and predictive performance across diverse patient populations (27, 28).

**Figure 1:**
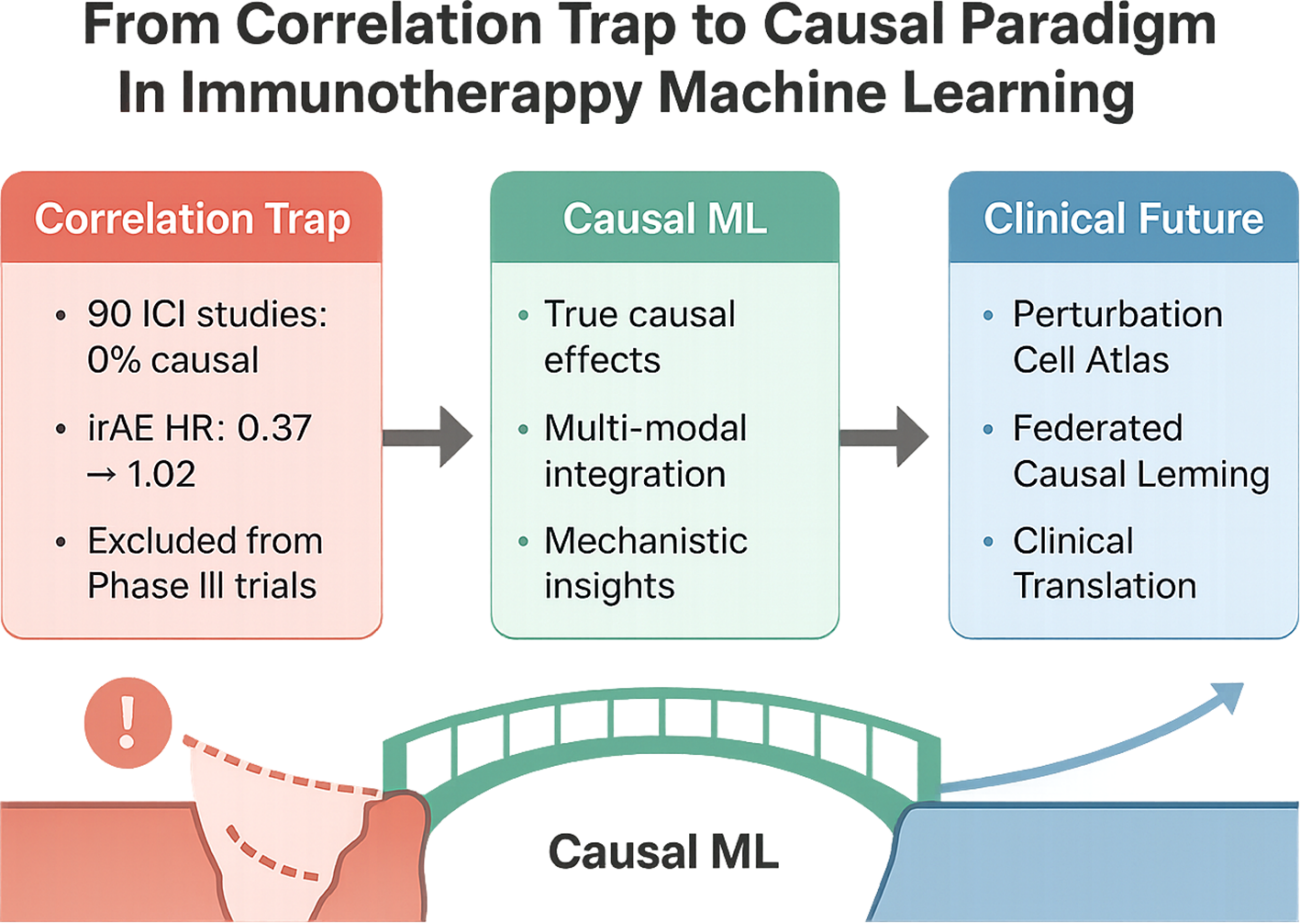
Transitioning from the correlation trap to the causal paradigm in immunotherapy machine learning. This figure illustrates the urgent need and conceptual roadmap for transitioning machine learning applications in immunotherapy research from correlation-based analyses to causal inference frameworks. The left red module highlights critical issues in current practice: among 90 ICI (immune checkpoint inhibitor) studies, none incorporated causal inference; the hazard ratio (HR) for immune-related adverse events (irAEs) shifted from 0.37 to 1.02 after causal bias correction, underscoring the misleading nature of pure correlational analysis. Moreover, some models were excluded from Phase III clinical trials due to a lack of causal validation. The central green bridge represents the solution offered by causal machine learning (Causal ML), characterized by three key strengths: identifying true causal effects, integrating multimodal data (genomics, imaging, and clinical records), and providing interpretable mechanistic insights. The right blue module envisions future breakthroughs over the next 5-10 years, including the development of the Perturbation Cell Atlas, federated causal learning approaches, and eventual clinical translation. The cliff–bridge–shoreline metaphor visually encapsulates the methodological leap required to shift from flawed analytics to a robust scientific paradigm.

This Perspective aims to systematically highlight the paradigm-shifting value of causal machine learning in immunological research. We focus on the following key questions (**Figure 1**):

1. **Pitfalls of correlation-based approaches**: Why do conventional models relying solely on correlation lead to conflicting conclusions? For instance, how should we reinterpret established “consensus” when the hazard ratio (HR) of immune-related adverse events (irAEs) for survival shifts from 0.37 to 1.02 after causal correction?
2. **Unique advantages of causal ML**: How does causal ML bridge the gap from “correlation discovery” to “causal identification”? What breakthrough capabilities does it offer in capturing the complexity of the immune system?
3. **Implementation challenges**: How do issues such as data quality, model interpretability, and interdisciplinary collaboration hinder the clinical adoption of causal ML?
4. **Future directions**: From “perturbed cellular atlases” to federated causal learning, which innovations over the next 5-10 years are most likely to translate causal ML from theory into real-world practice?

## 2 Misconceptions in immunological research: equating correlation with causation

In current immunotherapeutic research, traditional machine learning (ML) models primarily rely on retrospective data mining of correlations (29), yet they often fail to explore the underlying causal mechanisms (30). For instance, in studies on the gut microbiome and immune checkpoint inhibitors (ICIs), although advanced algorithms such as Random Forests and SVMs were employed, only 4 out of 27 studies conducted cross-validation. Furthermore, key confounding factors such as antibiotic use and dietary differences were not adequately controlled, resulting in highly heterogeneous and unreliable conclusions regarding the efficacy of the same microbial strains (31). Similarly, in the analysis of immune-related adverse events (irAEs) and survival, traditional Cox regression yielded a hazard ratio (HR) of 0.37, implying a protective effect of irAEs. However, causal ML using target trial emulation (TTE) to correct for immortal time bias revealed a true HR of 1.02—completely overturning the conventional belief that irAEs improve prognosis (32). These findings underscore the urgent need for sound causal inference in immunological studies to avoid conclusions that contradict biological plausibility.

Moreover, the insufficient recognition of the importance of causal inference among researchers (33) has led to multiple problems. Notably, effective therapies may be erroneously rejected due to improper grouping strategies (34), while correlations that appear statistically significant (35) may be misinterpreted as causal relationships (36), leading to misleading clinical implications (37). For example, studies examining the impact of antibiotic exposure on ICI outcomes reported a statistically significant HR of approximately 1.3, yet the authors explicitly acknowledged the presence of residual unmeasured confounders. This raises the risk of inappropriate clinical decisions, such as the unjustified discontinuation of antibiotics due to a presumed class-wide harmful effect (38). Likewise, deep learning models based on CT radiomics for predicting ICI responses reported an AUC of ~0.71, but the signal captured largely reflected confounders such as tumor burden and treatment line rather than true drug sensitivity, casting doubt on the validity of the model’s conclusions (39).

Therefore, neglecting causal inference not only compromises the reliability of study results (40), impedes clinical translation (41–43), and misguides clinical decision-making, but also wastes research resources and delays the development of effective therapies (44). A typical example is seen in COVID - 19 vaccine research, where including non-virus-related hospitalizations (“false-positive cases”) led to substantial underestimation of the protective effect of vaccines that primarily prevent severe post-infection complications rather than infection itself—ultimately resulting in misleading conclusions about vaccine efficacy (45).

Although the importance of causal inference has been increasingly recognized in clinical research, many studies still rely on conventional causal inference methods, which face significant challenges in practice. Randomized controlled trials (RCTs) are often infeasible due to high costs, ethical constraints, and heterogeneity among patients (46). Stratified designs in observational studies struggle with high-dimensional omics data, and multivariable regression fails to capture the nonlinear characteristics of the immune system (47). Propensity score methods (PSM), based on the unrealistic assumption that all confounders are measurable, have been misapplied in 72% of studies (8). Mendelian Randomization (MR) also faces methodological limitations, including susceptibility to false associations and estimation bias stemming from the quality of genetic instruments and core assumptions (48, 49). Specifically, MR applications in immunology face four major hurdles: violation of the instrumental variable assumption due to pleiotropy; weak instruments owing to low heritability of immune exposures; a mismatch between lifelong genetic effects and short-term therapeutic interventions; and systematic bias from population stratification (50–52). Collectively, these limitations have constrained the application and scalability of traditional causal inference approaches in immunology.

**Table 1** presents representative cases where correlation-based analyses failed, while **Table 2** summarizes the limitations of traditional causal inference methods.

**Table 1:** Representative bias cases in immune studies dominated by correlation-based machine learning.

Study & Year	Correlation-based ML/ statistical approach	Identified bias	Evidence	Ref.
Zhang et al., 2023	Cross-cohort Random Forest / SVM in microbiota-ICI response review	Antibiotic use, dietary/geographic differences, sequencing batch effects	Among 27 studies, only 4 cross-validated the same strains; conclusions showed high heterogeneity, possibly leading to the erroneous rejection or overpromotion of specific microbial therapies.	(31)
Pichler et al., 2025	Early studies used Cox/log-rank; this study used Target Trial Emulation (TTE)	Immortal time bias (irAEs occur only in survivors)	Conventional analysis showed HR = 0.37, but TTE-corrected HR = 1.02, overturning the “irAE improves prognosis” claim; without correction, irAE benefits may be exaggerated, misleading dose management.	(32)
Eng et al., 2023	Population database with Cox + PSM to assess antibiotic exposure on ICI outcomes	Confounding by infection severity, concomitant medications, baseline ECOG status	Although HR ≈ 1.3 was statistically significant, authors acknowledged "residual unmeasured confounding"; could mislead clinicians into believing all antibiotics are harmful, leading to inappropriate withdrawal.	(38)
Sako et al., 2024	3D ResNet + multitask deep learning for CT-based ICI efficacy prediction	Tumor burden, treatment line, imaging device heterogeneity	Reported AUC ≈ 0.71; authors emphasized the need for prospective validation, warning that tumor size/stage signals may be misinterpreted as drug sensitivity, affecting patient stratification.	(39)

ML, machine learning; ICI, immune checkpoint inhibitor; irAE, immune-related adverse event; HR, hazard ratio; TTE, target trial emulation; PSM, propensity score matching; ECOG, Eastern Cooperative Oncology Group performance status; AUC, area under the receiver operating characteristic curve.

This table presents representative cases from four high-impact areas of immunological research—microbiome, survival analysis, drug exposure, and radiomics—demonstrating how correlation-based approaches can lead to misleading interpretations when causal inference is neglected.

**Table 2:** Limitations of traditional causal inference methods in immune-related studies.

Traditional method	Core mechanism	Limitations	Ref.
Randomized Controlled Trial (RCT)	Eliminates both observed and unobserved confounding via random allocation	High cost and ethical concerns; significant patient heterogeneity; multi-arm RCTs are impractical for ICI combination and dynamic exposures	(11, 46)
Stratified/Blocked Design	Predefined stratification based on limited covariates	High-dimensional omics (>10^4^ features) leads to dimensionality explosion; residual confounding remains	(47, 100)
Multivariable Regression Adjustment	Uses linear/generalized linear models to control covariates	Requires correct model specification; immune nonlinearity and interaction effects are easily mis-specified	(47, 100)
Propensity Score Matching / Inverse Probability Weighting (PSM/IPW)	Balances observed covariates through a single score	Relies on the “no unmeasured confounding” assumption; unstable in high-dimensional settings; 72% of 90 ICI studies still rely on PSM or correlative ML, lacking prospective design	(8, 9)
Mendelian Randomization (MR)	Uses germline genetic variants as instrumental variables to mimic natural randomization	(1) Horizontal/related pleiotropy may violate exclusion restriction; (2) limited heritability of immune exposures → weak instruments; (3) lifetime average effects ≠ short-term drug effects; (4) population stratification and LD structure may introduce bias	(50–52)

RCT, randomized controlled trial; PSM, propensity score matching; IPW, inverse probability weighting; MR, Mendelian randomization; ICI, immune checkpoint inhibitor; LD, linkage disequilibrium.

This table highlights the key limitations of five classical causal inference approaches when applied to high-dimensional, nonlinear, and heterogeneous data settings in immunotherapy research.

## 3 Unique advantages of causal inference machine learning models

**Figure 2:**
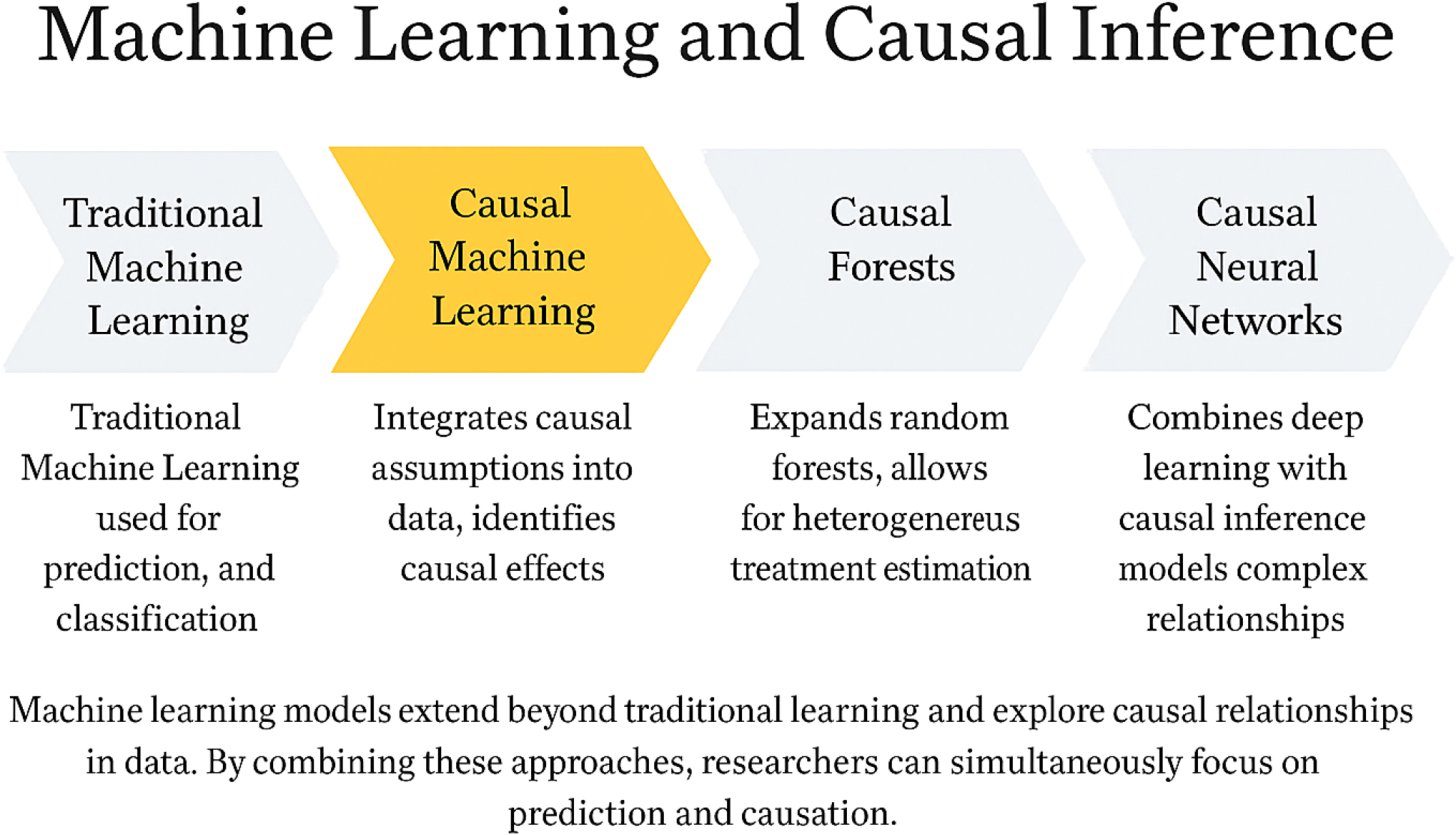
Integrating machine learning and causal inference: from predictive models to causal understanding. This figure illustrates the methodological evolution of machine learning from conventional predictive modeling toward causal inference. Traditional machine learning focuses on prediction and classification tasks without addressing underlying causal mechanisms. Causal machine learning integrates causal assumptions into data analysis to estimate true treatment effects. Causal forests extend random forests to enable estimation of heterogeneous treatment effects. Causal neural networks combine deep learning architectures with causal inference to model complex relationships. Together, these approaches bridge the gap between predictive accuracy and causal interpretability, providing a comprehensive analytical framework for immunotherapy research.

To overcome the limitations of both traditional causal inference and conventional machine learning approaches, causal inference-based machine learning (causal ML) models have emerged (**Figure 2**). Compared to classical causal methods such as propensity score matching (PSM), Cox regression, or linear models, causal ML lifts the constraints of strict parametric assumptions and rigid model forms, enabling more flexible modeling of the nonlinear dynamics and high-dimensional interactions inherent to immune systems (53–55). For instance, CV-TMLE, when applied in a small-scale study of only 168 ICU patients with COVID - 19, employed the Super Learner ensemble approach to effectively relax regularity conditions and increased the 95% confidence interval coverage by 10-20 percentage points compared to standard methods (53). Similarly, the ANN-DML estimator demonstrated a ~30% reduction in mean squared error (MSE) relative to conventional kernel smoothing methods when handling extremely high-dimensional scenarios where the number of immune biomarkers scales with sample size (p → 2n) (54).

Moreover, causal ML enables multi-modal modeling by integrating imaging, text, time-series, and genomic data. For example, Clinical Transformer can fuse clinical records, laboratory metrics, and sequencing data. By leveraging counterfactual perturbation strategies, it achieved an improvement of 0.05-0.10 in C-index across seven cancer types (56). MOFS effectively integrates MRI, pathology, and multi-omics data to identify glioma subtypes most responsive to anti-PD-1 therapy (57), while Bio-relevant AI combines imaging, pathology, and gene expression data to help 32% of stage II colorectal cancer patients avoid unnecessary chemotherapy (58). These unique strengths contribute to more accurate prediction of therapeutic outcomes (33), optimizing drug use and enhancing treatment efficacy (59).

In contrast to conventional machine learning methods such as random forests, LASSO, or deep learning—models that rely solely on correlational pattern discovery—causal ML shifts the focus from predicting associations to identifying causality. For instance, the Super Learner ITE framework estimates individual treatment effects (ITE) through model ensembling, achieving an AUC of 0.77 in external validation, with decision curve analysis showing a significantly higher net clinical benefit compared to treat-all or SAPS-II strategies (60). Similarly, in the MiCML platform study, Causal Forest utilized adaptive partitioning to estimate conditional average treatment effects (CATE), reducing prediction error for treatment–microbiome interaction effects by 25-40% compared to traditional LASSO regression (55).

Furthermore, causal ML effectively addresses key limitations of correlational models—namely spurious associations and confounding bias—by enabling robust control of unmeasured confounding (61). This facilitates the clarification of true causal relationships between immune cells and disease (36). For instance, COCA utilizes negative control outcome calibration to restrict estimation bias to less than 40% of that seen in conventional OLS models (62), and CV-TMLE improves 95% confidence interval coverage (53). Collectively, these advantages enhance model performance (63), clinical interpretability (43), and generalizability (64), providing robust scientific guidance for clinical decision-making (40).

In addition, mechanism-aware causal ML approaches embed biological prior knowledge into model structures, achieving a unification of data-driven and mechanism-driven strategies—a closed loop between computation and experimentation (65). This integration enables better capture of complex clinical phenotypes, deeper mechanistic insights (41), and enhanced feasibility and translational value of biomedical research (66). Consequently, causal ML provides promising avenues for early detection strategies (64) and novel drug development pipelines (34).

**Table 3** summarizes the unique advantages of causal ML methods, while **Table 4** outlines their applications in multi-dimensional data integration.

**Table 3:** Advantages of causal machine learning (causal ML) over traditional machine learning methods.

Causal ML method	Traditional method	Limitations of traditional method	Causal ML highlights	Dataset / Scenario	Performance gains	Ref.
CV-TMLE / DML	Logistic regression + PSM	Unstable estimation in high-dimensional covariates; model misspecification	Super Learner ensemble + targeted updates; doubly robust	COVID-19 ICU (n = 168): dexamethasone vs hydrocortisone on NLR	95% CI coverage ↑ by 10–20 percentage points; no bias increase	(53)
TMLE-SL / BART	Multivariate linear regression	Linear assumptions; manual interaction terms	Model-agnostic; tree-/network-based flexible estimation	COVID-19 ICU (n = 168): dexamethasone vs hydrocortisone on NLR	CI width ↓ ≈12%; more stable estimates	(101)
Super Learner ITE	SAPS-II rules / Treat-all	Ignores patient heterogeneity; low net benefit in decision curves	Direct ensemble estimation of individual treatment effects	4 septic shock steroid RCTs (training) + external RCT validation	External AUC = 0.77; net benefit > Treat-all/SAPS-II strategies	(60)
Causal Forest / X-Learner	Random forest / LASSO	Only average treatment effects; heterogeneity missed	Adaptive partitioning for CATE estimation	MiCML: gut microbiota + ICI response (n = 128)	Interaction effect error ↓ 25–40%	(55)
ANN-DML Estimator	Kernel/spline nonparametric	“Curse of dimensionality”	Neural net approximates both treatment and outcome models	Simulated immunomarker scenario with p → 2n	MSE ↓ ≈30%	(54)
COCA (Negative Control Calibration)	OLS / traditional sensitivity analysis	Unmeasured confounding not identifiable	Negative control outcome + doubly robust via Lavaan framework	Education intervention + inflammation markers (simulation + real)	Bias ≤ 40% of OLS estimate	(62)
Mechanistic Deep Learning	End-to-end CNN/LSTM	Lacks mechanistic interpretability; weak extrapolation	Embeds ODE tumor-immune dynamics	Mathematical tumor models + in vitro experiments	RMSE ↓ 25%; reproducible via wet-lab validation	(65)
TNDDR (DML + Cross-fitting)	Classic test-negative logit	High-dimensional covariates inflate SE	Doubly robust + cross-fitting	Québec health records (age ≥60): COVID-19 vaccine effectiveness	SE ↓ 26–67%; generalization improved	(102)

CV-TMLE, cross-validated targeted maximum likelihood estimation; DML, double machine learning; BART, Bayesian additive regression trees; ITE, individualized treatment effect; CATE, conditional average treatment effect; COCA, causal outcome calibration using negative control; OLS, ordinary least squares; TNDDR, test-negative design doubly robust estimator; CI, confidence interval; pp, percentage points; MSE, mean squared error; RMSE, root mean squared error; SE, standard error. Arrows: ↑ indicates increase/improvement; ↓ indicates reduction.

This table summarizes eight representative Causal ML approaches, demonstrating their superiority over traditional methods in terms of robustness, flexibility, and capacity to handle high-dimensional, heterogeneous, and nonlinear immunological data.

**Table 4:** Applications of multimodal causal ML: integrated modeling of imaging, omics, clinical, textual, and temporal data.

Study & Year	Data modalities	Methodological mechanism	Dataset / Application outcome	Ref.
Clinical Transformer, 2025	Clinical records + lab indices + DNA/RNA sequencing	Transformer + self-attention; uses in silico counterfactual perturbations to explore immune benefit subgroups	External validation across 7 cancer types; C-index ↑ 0.05–0.10; identified a cohort potentially benefiting from immunotherapy	(56)
MOFS Fusion Framework, 2025	MRI + digital pathology + genomics/transcriptomics/proteomics	Deep feature fusion with explicit tracking of "latent causal features"	Stratified 3 glioma subtypes; MOFS3 subtype most sensitive to anti–PD-1 therapy	(57)
MMF HCC, 2025	CT imaging + serum AFP / liver function / clinical phenotypes	Residual-attention fusion; quantifies ICI benefit via individualized OS/PFS counterfactual contrasts	C-index = 0.76 for ICI benefit prediction; HR = 2.44 in high-risk group	(103)
Bio-relevant AI (CRC II), 2025	CT radiomics + histopathology + gene expression + clinical stage	Joint image-omics embedding + counterfactual risk difference for chemotherapy selection	Helped 32% of stage II colorectal cancer patients avoid unnecessary chemotherapy; RA-net interpretability score ↑	(58)
CRISP (ICU), 2025	Temporal vital signs + clinical notes + lab tests	Native counterfactual generation + causal graph priors to address class imbalance	AUROC = 0.90–0.95 across 3 centers; cross-domain generalization error ↓ 15%	(104)

C-index, concordance index; OS, overall survival; PFS, progression-free survival; HR, hazard ratio; AUROC, area under the receiver operating characteristic curve; AFP, alpha-fetoprotein; MOFS, multi-omics feature selection; MMF, multimodal fusion; HCC, hepatocellular carcinoma; CRC, colorectal cancer; CRISP, causal risk prediction in ICU; ICU, intensive care unit. Arrows: ↑ denotes improvement/increase; ↓ denotes reduction.

This table illustrates how causal ML enables personalized treatment effect estimation by integrating multimodal data—spanning imaging, omics, clinical indicators, text, and temporal signals—across diverse immunotherapy-related scenarios.

## 3 Challenges in the application of causal inference machine learning models

At the data acquisition level, the presence of inaccurate or incomplete data significantly hinders the implementation of causal inference models. In particular, measurement errors can amplify causal bias, thereby undermining the reliability of results (67). Moreover, when missing data violate identifiability assumptions, no estimator can recover the true causal effect, rendering any derived causal inference invalid (68).

At the clinical application level, causal machine learning (Causal ML) models often exhibit a “black-box” nature, which severely limits clinician acceptance (69). When internal parameters and computational processes become overly complex, it becomes difficult for clinicians to understand how conclusions are derived, ultimately impeding clinical translation (70, 71).

At the research methodology level, both methodological selection difficulties and interdisciplinary collaboration barriers constrain the advancement of Causal ML in immunological research. Causal relationships vary in structure and often require tailored methods, yet the abundance of available approaches—each with unique limitations—makes optimal selection challenging, especially for researchers with limited formal training in causal modeling (33). Furthermore, interdisciplinary efforts are frequently impeded by cultural and conceptual gaps between domains. For instance, biomedical scientists tend to focus on clinical applicability, statisticians emphasize methodological validity, and computer scientists prioritize algorithmic performance. These differing priorities can lead to communication breakdowns and ultimately slow scientific progress (72, 73).

**Table 5** summarizes the three major challenges faced by Causal ML.

**Table 5:** Challenges and limitations in applying causal machine learning (causal ML) models in immunological research.

Challenge Category	Study focus	Key findings	Ref.
Data Collection: Inaccuracy and Missingness	HIV cohort: modeling measurement error and missingness as latent outcome missingness	“All your data are always missing” demonstrates that observational data alone cannot validate causal assumptions; measurement error → information loss → biased ATE; highlights need for additional assumptions or sensitivity analyses in high-noise immunological datasets	(67)
	Pediatric long-term medication study using graphical models to analyze missingness patterns	In longitudinal pharmacokinetic data, if missing nodes violate identifiability conditions, no estimator can recover the true causal effect; emphasizes prior design and imputation strategy	(68)
Clinical Application: Lack of Model Interpretability	Four clinical decision support scenarios comparing interpretable vs. non-interpretable models	Summarizes seven dimensions of interpretability (e.g., stakeholder type, transparency-accuracy trade-off); concludes that complex causal models lacking clear explanation are difficult to integrate into clinical workflows	(69)
	Review of Causal ML in precision medicine, including a section on explainability and regulatory barriers	While causal graphs and deep models can address interventional questions, lack of clinician-facing visualization and auditing tools sustains “black-box” concerns	(71)
Methodological & Cross-Disciplinary Barriers	Systematic evaluation of causal inference strategies (IV, RD, PS, G-methods); introduces “evidence triangulation”	Many researchers lack training to choose appropriate methods; recommends triangulation and cross-disciplinary validation for greater robustness	(105)
	Commentary on clinical research practice	Cultural divides between epidemiology, statistics, and clinical medicine hinder communication; advocates for a unified vocabulary and collaborative platforms—still a major bottleneck in immunological causal research	(72)

ATE, average treatment effect; HIV, human immunodeficiency virus; IV, instrumental variable; RD, regression discontinuity; G-methods, graphical-based causal inference methods; HTA, health technology assessment.

This table synthesizes three major classes of challenges faced when applying causal ML models to immunological studies: (1) data quality and missingness, (2) model interpretability and clinical adoption, and (3) methodological complexity and interdisciplinary barriers.

## 4 Discussion

Over the next five years, addressing the two core challenges—data quality and model interpretability—will require the development of innovative technical solutions. In terms of data quality, the integration of multiple imputation with the G-formula has significantly reduced bias caused by missingness in cystic fibrosis studies (74). Likewise, the MI-BART method has demonstrated superior robustness in multi-treatment comparisons (75), offering promising prospects for enhanced data control and quality improvement over the next 5-10 years.

Regarding interpretability, studies have shown that Causal-XAI hybrid frameworks can generate causal attribution heatmaps, enabling physicians to better understand image-based decisions (76). In addition, CLARUS, an interactive counterfactual reasoning platform, allows clinical experts to directly manipulate and verify model reasoning chains (77). This effectively addresses the “black-box” issue by clarifying causal pathways underlying model outputs (78), thereby improving both clinical decision-making and regulatory trust, ultimately facilitating clinical translation (79). In the future, the integration of Bayesian nonparametric models and natural language processing (NLP) is expected to further enhance model performance by extracting authentic causal structures from large-scale biomedical data (80), revealing deep causal relationships and identifying novel therapeutic targets (81, 82).

In the next 5-10 years, methodological integration will become a central theme. The emerging “triangulation framework” will be more widely adopted. This framework enhances the robustness of causal inference by integrating and cross-validating multiple approaches such as instrumental variables (IVs), regression discontinuity (RD), and propensity scores (83). In parallel, strengthening interdisciplinary collaboration and talent development will become essential. Multidisciplinary teams can develop shared terminologies and workflows, promoting effective integration across epidemiology, economics, and clinical medicine (84) and enabling each field to contribute its strengths to solve complex problems (42, 73). Cultivating versatile professionals capable of navigating the intricacies of immune-related biological systems (85) will help dismantle disciplinary silos and address challenges in resource allocation and coordination (86). This integrated approach will enable more comprehensive solutions (87) to meet the rapidly evolving demands of immune drug research (88). Furthermore, academic institutions should establish dedicated programs and curricula to train cross-disciplinary talent in causal inference and immunotherapy (42, 73), fostering the convergence of modern science and specialized education (89), promoting skills development (90), and facilitating global collaboration in immunology research (91), injecting new vitality and opportunity into the field.

In the next 5-10 years, causal inference models are expected to be widely implemented in clinical immunology. One notable development is the “Perturbation Cell Atlas” proposed by Rood et al., which represents a conceptual turning point. Future research will likely build on this by leveraging large-scale CRISPR-scRNA-seq perturbation datasets to train and deploy foundational causal models for practical guidance (92). Technologically, tools such as Velorama, which has shown great promise in immune differentiation studies, will play a pivotal role. By integrating RNA velocity to express cellular developmental trajectories as directed acyclic graphs (DAGs), these tools enable causal network inference at single-cell resolution, a capability expected to be expanded in future research (93).

With the continued advancement of artificial intelligence, AI-assisted vaccine design is poised to become a prevailing trend. This will necessitate the use of target trial emulation, causal NLP, and federated causal estimation frameworks to identify causally relevant endpoints and accelerate critical discoveries (94, 95). Moreover, as federated learning frameworks mature across institutions (76), interpretable causal tools such as CIMLA will likely become standardized (96), enabling a full transition of causal inference from theoretical development to routine clinical decision support. This process will be further facilitated by improvements in data quality and model robustness through rigorous control of covariates and confounding variables (97–99), which are essential for enhancing the credibility, transparency, and real-world applicability of causal models in clinical settings.

**Table 6** presents strategies to address the three major challenges, while **Table 7** outlines the projected applications of causal ML in immunology over the next 5-10 years.

**Table 6:** Technical strategies addressing the three core challenges in causal machine learning (causal ML) applications.

Challenge	Solution strategy	Supporting evidence	Ref.
Poor Data Quality (Missingness / Measurement Error)	Multiple Imputation + G-formula: Integrates Bayesian multiple imputation into G-formula to simultaneously estimate counterfactuals and impute missing data	Demonstrated in cystic fibrosis cohort to recover time-varying treatment effects and significantly reduce bias from missing data	(74)
	MI-BART / GAM for multiple-treatment scenarios: Fits flexible models for each treatment arm and imputes all counterfactual outcomes	MI-BART showed superior robustness and CI coverage over weighting/matching in multi-center readmission risk comparisons	(75)
Limited Clinical Interpretability	Causal-XAI federated learning: Employs causal sparsity weights and blockchain validation for federated clinical feature attribution	Heterogeneity-aware causal sparsity FL reduced communication cost (↓) and improved performance (↑), generating causal heatmaps interpretable to clinicians	(76)
	CLARUS: An interactive counterfactual explanation platform allowing clinicians to explore GNN-based reasoning chains	Combines manual and automated interfaces; enables expert manipulation and validation of reasoning paths to enhance model trust	(77)
Difficulty in Method Selection	Evidence triangulation: Parallel use of IV, RD, propensity scores, and G-methods with cross-validation across designs	Proposes “biased-but-directionally-consistent cross-validation” framework, emphasizing method and cohort consistency as a criterion for causal reliability	(83)
Interdisciplinary Collaboration Barriers	Integration of Health Decision Science & Causal Inference: Aligns DAGs and decision models under shared HTA frameworks	Highlights complementarity of causal inference and economic evaluation; provides joint workflow templates for epidemiology, economics, and clinical collaboration	(84)

MI-BART, multiple imputation with Bayesian additive regression trees; GAM, generalized additive model; XAI, explainable artificial intelligence; FL, federated learning; GNN, graph neural network; DAG, directed acyclic graph; HTA, health technology assessment. Arrows: ↑ indicates enhancement/improvement; ↓ indicates reduction.

This table presents validated methodological solutions corresponding to the three core Causal ML challenges—data quality, interpretability, and method selection—supported by empirical evidence and interdisciplinary integration practices.

**Table 7:** Future directions for causal machine learning in immunology over the next 5–10 years.

Research direction	Theoretical foundation	Ref.
Large-scale Perturbation Single-Cell Atlas	Introduced the concept of a “Perturbation Cell Atlas,” proposing training generative causal models on millions of CRISPR-based scRNA-seq perturbation profiles	(92)
Causal GRN Inference from Single-Cell Data	Velorama applies RNA velocity to represent developmental trajectories as DAGs, enabling inference of fast/slow transcriptional regulators and dynamic GRNs at single-cell resolution	(93)
Explainable Causal AI for Multi-omics Integration	CIMLA framework combines SHAP-style interpretability with structural causal models to trace directional regulation in immune-metabolic pathways	(96)
Causal Inference in Vaccine Design	AI-driven vaccinology requires target trial emulation and causal NLP to accelerate discovery of protective correlates	(94)
Toolchains for Single-Cell Causal Discovery	CausalCell platform integrates multiple algorithms to facilitate causal signal mining in single-cell immunology	(106)
Explainable Federated Causal Learning	Combines causal sparsity weighting with blockchain-based data quality control to enable multi-institutional causal modeling under privacy-preserving constraints	(76)
Cross-Site Distributed Causal Inference	Federated causal estimation will extend to Cox and Aalen–Johansen models, correcting for heterogeneity across clinical sites	(95)

CRISPR, clustered regularly interspaced short palindromic repeats; scRNA-seq, single-cell RNA sequencing; GRN, gene regulatory network; DAG, directed acyclic graph; SHAP, Shapley additive explanations; NLP, natural language processing.

This table highlights emerging trajectories in causal ML for immunological research, ranging from single-cell network inference to federated causal modeling, paving the way for more interpretable, scalable, and collaborative approaches in precision immunology.

## Data Availability

The original contributions presented in the study are included in the article/supplementary material. Further inquiries can be directed to the corresponding author.
